# Preliminary result of 4 years of conservative management Juvenile Idiopathic Scoliosis, case report

**DOI:** 10.1186/1748-7161-8-S2-O47

**Published:** 2013-09-18

**Authors:** Andrea Lebel

**Affiliations:** 1 Ottawa & District Physiotherapy Clinic, Ottawa, Canada

## Background

Based on Scoliosis Research Society guidelines, individuals with JIS over 50 degrees Cobb are usually treated surgically. The JIS surgery option in Ottawa, Canada, is the growing-rod surgery, extended every 6 months until end of growth. Multiple surgery complication rate is high (57%) in this age group. On the other hand, there is a lack of evidence for the effectiveness of conservative management of JIS. 

## Methods

In 2008, a 5-year-old girl was diagnosed with JIS: 53 degrees thoracic and 24 degrees lumbar curve, with a risk of progression calculated at 90-100%. The patient started Schroth physiotherapy in 2009 in combination with full-time bracing in an outpatient setting, was initially seen once a week, then monitored regularly every 3-6 months. The patient’s mother was instructed on the home exercise program that was designed specifically for the patient’s curve pattern, following the principals of the Schroth method. The girl was followed for four years. During this time, objective data was collected: x-ray, ATR, Chest extension, Vital Capacity (V/C) and clinical pictures. 

## Results

Since 2011, the patient was showing improvements in Cobb angle and ATR: out-of-brace x-ray measured 20 degrees in 2011 and in 16 degrees in 2012. ATR in 2009 was 18 degrees and reduced to 5 degrees in 2013. Chest expansion improved from 1 cm in 2009 to 7cm in 2012. Her V/C and lung development in 2012 were found to be normal for her age and height (2000 ml). The patient’s height measurement increased 27 cm, from 110cm in 2009 to 137cm 2013, and she had a normal activity level. Other benefits of this treatment method included minimal x-ray exposure, no hospitalization or frequent medical appointments for pre- and post-surgery check-ups (patient avoided 8-9 growing rod surgeries so far). 

## Conclusions and discussion

Even though the Schroth method is questionable for a patient as young as 5 years old, in this case report, the patient was able to carry out Schroth exercises at home with supervision by her mother and showed consistent and significant improvements over the years of the treatment. In general, this JIS patient is responding well to Schroth physiotherapy and full-time bracing. Conservative management for JIS, including bracing and physiotherapy in an outpatient setting, could be effective and should be offered to the patient and family as an option when treating JIS. 
